# Disparity in Mpox awareness and vaccine hesitancy: a cross-sectional study of 3,483 university students in Northwest China

**DOI:** 10.3389/fpubh.2025.1657497

**Published:** 2025-11-27

**Authors:** Ying Zhang, Jun Gan, Dong Miao, Ning-Hui Zhu, Li Xiao, Qing-Ming Shi, Yue Liu, Yuan-Yuan Li, Xiao-Bing Hu, Jun-Feng Zheng, Wei Wang, Ji-Jun Chen, Zi-Peng Li, Jing-Jing Yu, Wen-Juan Ba, Yi-Jun Yang, Jing Zuo, Xiao-Ning Wang, Xiao-Lei Ye

**Affiliations:** 1 The First Affiliated Hospital of Xi'an Jiaotong University, Xi'an, Shaanxi, China; 2 Center for Disease Control and Prevention in Western Theater Command, Lanzhou, Ganshu, China; 3 The 980th Hospital of the Joint Logistics Support Force of the PLA, Shijiazhuang, Hebei, China; 4 The Second Affiliated Hospital of Xi'an Jiaotong University, Xi'an, Shaanxi, China; 5 Department of Human Anatomy and Developmental and Regenerative Biology Laboratory, Chengdu Medical College, Chengdu, Sichuan, China; 6 Lanzhou Center for Disease Control and Prevention, Lanzhou, Ganshu, China

**Keywords:** Mpox, university students, knowledge disparity, vaccine hesitancy, northwestern China

## Abstract

**Background:**

Mpox has shown a westward spreading trend in China and university students are a high-risk group. This study aimed to assess the current status of Mpox-related knowledge, attitudes, vaccination willingness, and associated factors among college students in northwestern China, so as to provide a basis for formulating targeted prevention and control strategies.

**Methods:**

A cross-sectional survey utilizing a structured questionnaire was administered to students from nine higher education institutions in northwestern China between October 1 and 14, 2024.

**Results:**

The study sample comprised 3,483 university students, of whom 56.90% were female, 84.81% identified as Han ethnicity, 60.03% resided in rural areas, 52.89% were younger than 20 years, and 57.45% were enrolled in medical-related majors. The median Mpox knowledge score (Kscore) was 10, with only 33.68% classified into the high-score group (>11). Kscore was significantly higher among female, medical major, and urban residents, whereas smokers and alcohol consumers exhibited lower scores (all *p* < 0.001). Logistic regression identified medical major [adjusted odds ratio (aOR) = 1.336] and Han ethnicity (aOR = 1.242) as protective factors associated with higher Kscore, while male gender (aOR = 0.808), rural residence (aOR = 0.847), and alcohol consumption (aOR = 0.739) were risk factors. Vaccine acceptance was reported by 81.94% of participants, and side effects were primary concern for hesitancy. Female gender (aOR = 0.665), younger age (<20 years; aOR = 2.169), and heterosexual orientation (aOR = 2.835) were associated with greater willingness to receive vaccination. Spearman correlation analysis revealed significant positive correlations between Kscore and proactive information-seeking (*r* = 0.235, *p* < 0.001), vaccination willingness (*r* = 0.148, *p* < 0.001), and healthcare-seeking behavior (*r* = 0.146, *p* < 0.001).

**Conclusion:**

College students in northwestern China have insufficient Mpox knowledge but high vaccination acceptance. Targeted health education interventions should be implemented via new media platforms, focusing on male students, rural residents, and alcohol consumers, to enhance understanding of transmission routes and vaccine safety, reduce stigma, and strengthen Mpox prevention and control among young people in northwestern China.

## 1 Introduction

Mpox is a re-emerging infectious zoonotic disease caused by the monkeypox virus (MPXV), belonging to genus *Orthopoxvirus*, first identified in laboratory monkeys in 1958 (1). The first documented human infection occurred in 1970 in the Democratic Republic of the Congo. Historically, MPXV transmission was confined to Central and West Africa, with sporadic outbreaks reported globally prior to 2022. During this period, zoonotic spillover was the main transmission route, with limited human-to-human spread, and Clade I was the dominant strain in endemic regions.

A critical epidemiological shift began in May 2022, when Clade II-associated MPXV cases were continuously reported in non-endemic regions across Europe and Asia, including India, Singapore, and China, etc. (2). Most cases had a history of traveling to endemic areas or exposure to Mpox individuals. Notably, the Clade IIb subclade, recently designated “hMPOXV,” exhibits milder symptoms and lower mortality than Clade I but higher transmissibility, enabling sustained human-to-human spread. A systematic review identified sexual contact among men who have sex with men (MSM), household contact, and travel to endemic regions as significant risk factors for Mpox infection (1). As of August 2024, the WHO reported over 100,000 laboratory-confirmed cases reported leading to 220 deaths across 120 countries (3), resulting in Public Health Emergency of International Concern in 2022 and 2024 to the disease’s scale and geographic spread.

In mainland China, the first imported case was identified in Chongqing in 2022 (4, 5), followed by consecutive imported and local cases in Beijing (6, 7), Hunan (8) and Guangdong (9, 10). There were cumulative 2,984 confirmed cases from more than 30 province across China until 2025 (11). Mpox is a significant public health risks driven by its large MSM community and recovering international trade in China (12). In response, China classified Mpox as a Class B infectious disease in 2022. National Health Commission timely issued control and diagnosis/treatment guidelines (2022 version), relevant public prevention guideline (2023 version) was consecutively published to strengthen protection and curb domestic transmission.

Public awareness is critical for Mpox prevention, but most existing studies focused on Mpox knowledge, attitudes, and vaccine acceptance targeted to specific groups in China, such as HIV patients (13), medical workers (14) or the homosexual of MSM community (15, 16). In contrast, limited attention has been directed college students, which is one of the most densely population in China. Notably, sexual minority groups within Chinese college students population are at an elevated risk of engaging in high-risk sexual behaviors (17). According to a result of multicenter observational study in China, people with a college degree or higher account for 62.0% of Mpox patients which indicated college students was a high risk group of Mpox (18). Some studies have been conducted among college students located in southern (19), north and northeast China (20) to assess the knowledge and attitude. Currently, the epidemic is mainly distributed in the developed eastern regions, but there is a trend of spreading towards the western regions. Low-level cluster distributions have already emerged in places like Shaanxi, Gansu and Qinghai province (21). Research focusing on college students in western China remains scarce. Given the disparities in economic development, education, and social openness between western China and other regions, assessing the knowledge and vaccination willingness of college students in the western region is essential for informing targeted policy development.

To address this gap, we conducted a cross-sectional self-administered questionnaire study among college students in northwestern China to assess their knowledge and perceptions related to Mpox symptoms, transmission, prognosis, treatment, and vaccination willingness. This study aims to facilitate rapid and effective responses to potential future disease outbreaks.

## 2 Methods

### 2.1 Study design

**Figure 1:**
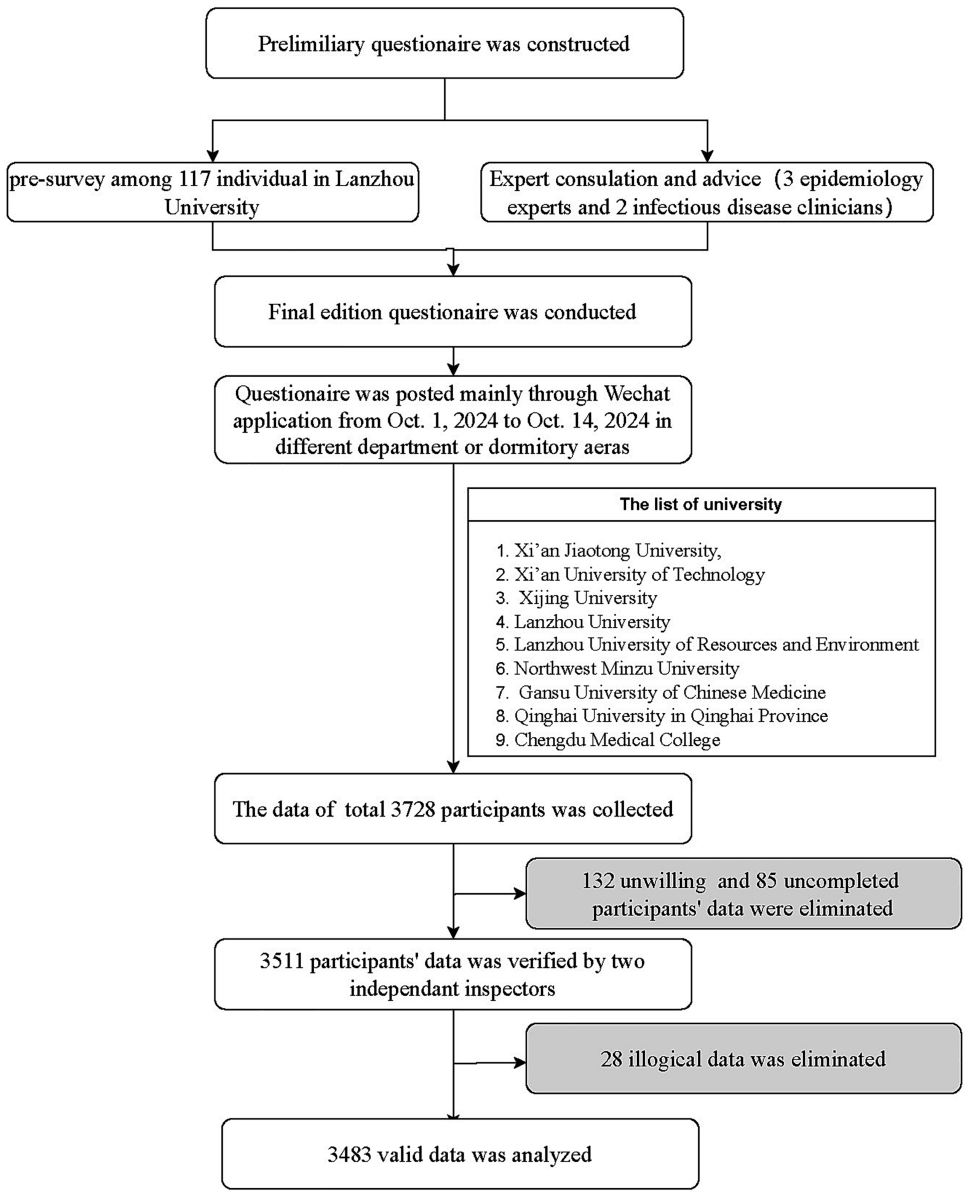
The detail process of rapid cross sectional questionnaire survey.

Between October 1 and 14, 2024, a rapid cross-sectional survey was conducted in northwestern China to assess university students’ knowledge and perceptions regarding Mpox. The questionnaire was initially developed through a comprehensive review of existing literature (13, 19, 22, 23) and consultation of pertinent guidelines, followed by refinement via pilot testing and iterative 5-person expert panel feedback. The finalized version was administered using an online survey platform PowerCX^1^ via a QR code and web link (Figure 1).

Data collection adopted convenience sampling with online-offline strategies. Student volunteers disseminated the survey links via WeChat Moments and posted QR codes on campus bulletin boards in different department or dormitory areas to avoid proximity bias. The study encompassed nine different types of universities across northwestern China (Figure 1). Eligible participants were college students aged ≥14 years who demonstrated comprehension of the survey and provided informed consent. Ethical approval for the study was granted by the Ethics Committee of the Center for Disease Control and Prevention in the Western Theater Command.

### 2.2 Informed consent and questionnaire design

At the commencement of the questionnaire, participants were provided with informed consent information. The questionnaire comprised three distinct sections as shown in Supplementary Table 1. The first section gathered demographic information, including variables such as gender, age, ethnicity, life behaviors, etc. The second section assessed participants’ knowledge through 15 items addressing etiology, modes of transmission, clinical symptoms, treatment options, and preventive measures; responses were recorded as “Yes,” “No,” or “Unclear.” The third section evaluated attitudes via 12 items measuring perceptions of protective behaviors, willingness to receive vaccination, and intentions to seek healthcare, utilizing a three-point Likert scale (“Agree,” “Disagree,” “Uncertain”). The instrument demonstrated excellent internal consistency, with a Cronbach’s alpha coefficient of 0.987, and its validity was established through expert consensus.

### 2.3 Statistical analyses

#### 2.3.1 Sample size estimation

The sample size was calculated using the single-sample proportion formula n (number) = [(Z_α/2_)^2^ *P* (1 − *P*)]/d^2^. Since no prior data existed on Mpox knowledge among university students in northwestern China, we set *P* at 50% to obtain a conservative, maximized estimate. A 95% confidence interval (Z_α/2_ = 1.96) and 5% margin of error (*d* = 0.05) yielded a minimum required sample size (n) of 384.

#### 2.3.2 Scoring of knowledge assessment

Each item assigned one point for correct response, resulting in a maximum knowledge score (Kscore) ranging from 0 to 15 (Supplementary Table 1). Based on predefined criteria, The cutoff value was set at 70% or higher of adequate score (24). Kscore >11 were classified into the high-score group, others were designated as the low-score group.

#### 2.3.3 Data verification and analysis

The data verification and cleaning were conducted by two independent researchers. The continuous variables conforming to the normal distribution were presented by means and standard deviation (mean ± SD), otherwise median and interquartile (median, IQR). Classification variables were expressed as frequency and percentage. The differences in Kscore among subgroups were examined using non-parametric tests. Spearman correlation was used to evaluate the correlation among Kscore, epidemic concerns, vaccination willingness, healthcare-seeking behavior, and proactive knowledge acquisition. Univariable and multivariable binary logistic regression were used to analysis the associated factors. A two-sided test was performed with *α* = 0.05. Data analysis and visualization was realized by IBM SPSS Statistics 25 and GraphPad Prism 8.

## 3 Results

### 3.1 Characteristics of participants

The survey was conducted over a two-week period, during which 3,728 individuals accessed the questionnaire link. A total of 132 individuals declined to participate, and 85 did not complete the survey, resulting in a response rate of 94.18%. After excluding 28 illogical questionnaires, the final dataset comprised 3,483 valid responses (Figure 1).

A total of 1,501 participants were male, representing 43.10% of the total respondents. Participants’ ages ranged from 16 to 32 years, with a mean age of 19.78 years (SD = 3.72). A total of 1,842 individuals (52.89%) were under 20 years of age, while 104 participants (2.99%) were aged 25 years or older. Educational attainment was classified into three categories: below bachelor’s degree (22.83%), bachelor’s degree (70.49%), and above bachelor’s degree (6.69%). Other detailed characteristics information was shown in Table 1.

**Table 1:** Demographic and general characteristics of participants (*N* = 3,483).

Characteristics		Number (%)	Mean ± SD
Gender	Males	1,501(43.10)	
	Females	1982(56.90)	
Age, years	Age<20	1842(52.89)	19.78 ± 3.72
	20 ≤ age<25	1,537(44.13)	
	Age≥25	104(2.99)	
Ethnicity	Han ethnicity	2,954(84.81)	
	Others	529(15.19)	
Educational level	Below bachelor degree	795(22.83)	
	Bachelor degree	2,455(70.49)	
	Above bachelor degree	233(6.69)	
Major	Medical	2001(57.45)	
	Non-medical	1,482(42.55)	
Family residence	Rural	2091(60.03)	
	Urban	1,392(39.97)	
Sexual orientation	Heterosexual	3,165(90.87)	
	Homosexual	68(1.95)	
	Bisexual	149(4.28)	
	Others	101(2.90)	
History of sexual intercourse	Yes	340(9.76)	
	No	3,143(90.24)	
History of multiple sexual partners	Yes	48(1.38)	
	No	3,435(98.26)	
History of SIDs	HIV	1(0.03)	
	Gonorrhea, syphilis, etc.	2(0.06)	
	Both	15(0.43)	
	Neither	3,465(99.48)	
Smoking	Yes	242(6.95)	
	No	3,241(93.05)	
Drinking	Yes	626(17.97)	
	No	2,857(82.03)	

### 3.2 Assessment of knowledge towards Mpox

#### 3.2.1 Awareness of Mpox-related key information

**Figure 2:**
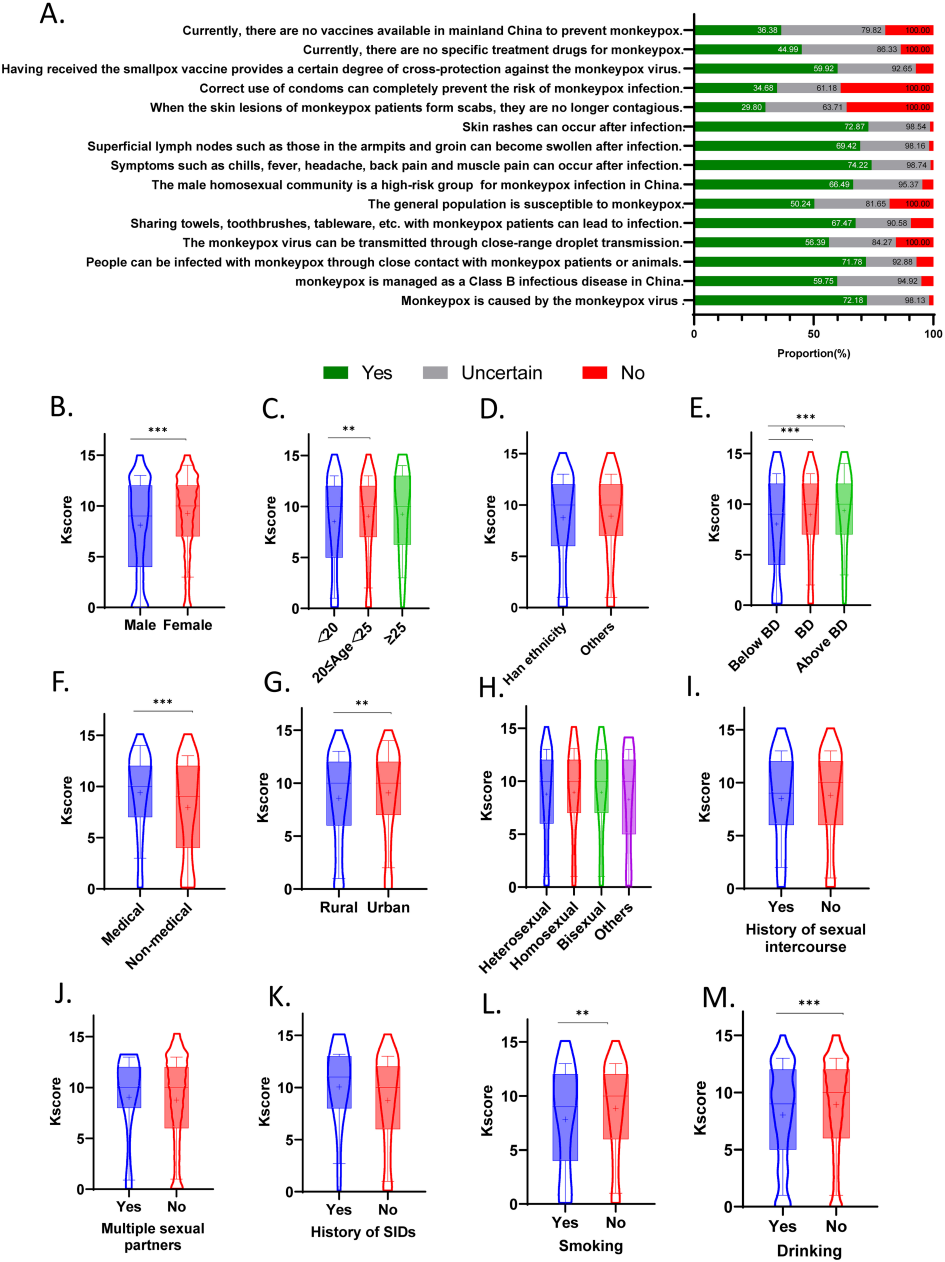
The results on knowledge section toward Mpox among 3,483 participants. **(A)** Response distribution for each question in the knowledge assessment. **(B–M)** The differences in knowledge scores among subgroups. The violin plots represent the overall data distribution, while the box plots indicate the 10th, 25th, 50th (median), 75th, and 90th percentiles. The mean values are denoted by “+.” **p < 0.01. ***p < 0.001.

The distribution of correct responses to 15 knowledge items is presented in Figure 2A. A total of 2,514 individuals (72.18%) accurately identified the MPXV as the etiological agent responsible for Mpox. Concerning public health management, 59.75% recognized that Mpox is designated as a Category B infectious disease in China. For transmission routes, 71.18% acknowledged close contact with infected individuals or animals as a mode of spread, 67.47% understood that sharing towels with infected patients may facilitate transmission, and only 56.39% correctly identified respiratory droplets as a potential route. General susceptibility to Mpox was known by 50.24% of respondents. 66.49% correctly identified MSM as the current high-risk group in China. For clinical manifestation, 74.22% recognized systemic symptoms (e.g., fever, headache), 72.87% were aware of cutaneous manifestations (e.g., rash), and 69.42% identified axillary or inguinal lymphadenopathy as a typical sign. Notably, only 36.29% correctly understood that infectivity persists during the scab-phase. Moreover, 38.82% acknowledged that condom use during sexual activity does not fully prevent transmission. Regarding therapeutic and preventive measures, 44.99 and 36.38% were aware of the current absence of Mpox-specific antivirals and vaccines in China.

#### 3.2.2 Distribution of knowledge scores and subgroup differences

The Kscore ranged from 0 to 15, with a median of 10 (IQR: 6–12). 1,173 (33.68%) participants were classified into the high-score group (>11), and 2,310 (66.32%) into the low-score group (≤11).

Subgroup analyses revealed that females attained a significantly higher Kscore compared to males (*p* < 0.001) (Figure 2B). Participants aged 20–25 years demonstrated higher Kscores than those under 20 years (*p* < 0.01) (Figure 2C). Individuals pursuing a bachelor’s degree (*p* < 0.001) or higher education (*p* < 0.001) scored significantly better than others (Figure 2E). Medical students achieved higher Kscore than non-medical counterparts (*p* < 0.001) (Figure 2F). Additionally, urban residents scored significantly higher than rural residents (*p* < 0.01) (Figure 2G). Conversely, smokers (*p* < 0.01) or alcohol consumers (*p* < 0.001) exhibited lower Kscore compared to non-smokers and non-drinkers, respectively (Figures 2L,M). No statistically significant differences in Kscore were observed among subgroups stratified by ethnicity, sexual orientation, sexual activity history, multiple sexual partners, or history of sexually transmitted infections (Figures 2D,H,I–K).

#### 3.2.3 Factors associated with high knowledge scores

To further clarify the determinants associated with achieving higher knowledge scores, both univariable and multivariable logistic regression analyses were conducted. Detailed findings are summarized in Table 2. Initial univariable analyses revealed several associated factors with high Kscore, such as male gender (OR: 0.867, 95% CI: 0.818–0.919), below bachelor’s degree (vs. above bachelor’s; OR: 0.738, 95% CI: 0.545–1.000), medical majors (OR: 1.250, 95% CI: 1.146–1.365), rural residents (OR: 0.903, 95% CI: 0.830–0.982), alcohol consumption (OR: 0.739, 95% CI: 0.607–0.901) and smoking (OR: 0.980, 95% CI: 0.962–0.998).

**Table 2:** The risk factor analysis of Knowledge score among 3,483 participants.

Variables	Total	High score group	Low score group	Univariate analysis		Multivariate analysis	
	N (%)	N (%)	N (%)	OR (95%*CI*)	*P*	aOR (95%*CI*)	*P*
Gender							
Male	1,501(43.10)	441(29.38)	1,060(70.62)	0.867(0.818 ~ 0.919)	**<0.001**	0.808(0.692 ~ 0.943)	**0.007**
Female	1982(56.90)	732(36.93)	1,250(63.07)	Reference		Reference	
Age, years					0.078		
Age<20	1842(52.89)	593(32.19)	1,249(67.81)	0.701(0.468 ~ 1.049)	0.084		
20 ≤ age<25	1,537(44.13)	538(35.00)	999(65.00)	0.795(0.530 ~ 1.192)	0.267		
Age≥25	104(2.99)	42(40.38)	62(59.62)	Reference			
Ethnicity							
Han ethnicity	2,954(84.81)	1,006(34.06)	1948(65.94)	1.101(0.929 ~ 1.304)	0.265	1.242(1.014 ~ 1.521)	**0.036**
Others	529(15.19)	167(31.57)	362(68.43)	Reference		Reference	
Educational level					0.121		
Below bachelor degree	795(22.83)	249(31.32)	546(68.68)	0.738(0.545 ~ 1.000)	**0.050**		
Bachelor degree	2,455(70.49)	835(34.01)	1,620(65.99)	0.834(0.632 ~ 1.100)	0.199		
Above bachelor degree	233(6.69)	89(38.20)	144(61.80)	Reference			
Major							
Medical	2001(57.45)	745(37.23)	1,256(62.77)	1.250(1.146 ~ 1.365)	**<0.001**	1.366(1.172 ~ 1.592)	**<0.001**
Non-medical	1,482(42.55)	428(28.88)	1,054(71.12)	Reference		Reference	
Family residence							
Rural	2091(60.03)	672(32.14)	1,419(67.86)	0.903(0.830 ~ 0.982)	**0.018**	0.847(0.733 ~ 0.978)	**0.024**
Urban	1,392(39.97)	501(35.99)	891(64.01)	Reference		Reference	
Sexual Orientation					0.540		
Heterosexual	3,165(90.87)	1,065(33.65)	2,100(66.35)	0.773(0.516 ~ 1.160)	0.214		
Homosexual	68(1.95)	22(32.36)	46(67.65)	0.729(0.382 ~ 1.391)	0.338		
Bisexual	149(4.28)	46(30.87)	103(69.13)	0.681(0.401 ~ 1.156)	0.155		
Others	101(2.90)	40(39.60)	61(60.40)	Reference			
History of sexual intercourse							
Yes	340(9.76)	107(31.47)	233(68.53)	0.989(0.967 ~ 1.012)	0.365		
No	3,143(90.24)	1,066(33.92)	2077(66.08)	Reference			
History of multiple sexual partners							
Yes	48(1.38)	16(33.33)	32(66.67)	1.000(0.992 ~ 1.008)	0.959		
No	3,435(98.62)	1,157(33.68)	2,278(66.32)	Reference			
History of SIDs							
Yes	18(0.52)	7(38.89)	11(61.11)	1.001(0.996 ~ 1.006)	0.639		
No	3,465(99.48)	1,166(33.65)	2,299(66.35)	Reference			
Smoking							
Yes	242(6.95)	67(27.69)	175(72.31)	0.980(0.962 ~ 0.998)	**0.041**		
No	3,241(93.05)	1,106(34.13)	2,135(65.87)	Reference			
Drinking							
Yes	626(17.97)	169(27.00)	457(73.00)	0.937(0.909 ~ 0.967)	**<0.001**	0.739(0.607 ~ 0.901)	**0.003**
No	2,857(82.03)	1,004(35.14)	1853(64.86)	Reference		Reference	

Hosmer- Lemeshow test: χ^2^ = 7.324, *P* = 0.396. *p* < 0.05 was presented with bold.

Subsequent multivariable analysis was performed to adjust for potential confounding variables, the Hosmer-Lemeshow test indicated an adequate model fit (χ^2^ = 7.324, *p* = 0.396). The adjusted results indicated that Han ethnicity [adjusted OR (aOR): 1.242, 95% CI: 1.014–1.521] and enrollment in medical majors (aOR: 1.336, 95% CI: 1.172–1.592) served as protective factors for achieving high knowledge scores. In contrast, male gender (aOR: 0.808, 95% CI: 0.692–0.943), rural family residence (aOR: 0.847, 95% CI: 0.733–0.978), and alcohol consumption (aOR: 0.739, 95% CI: 0.607–0.901) were identified as risk factors negatively associated with high knowledge scores (Table 2).

### 3.3 Assessment of Mpox-related attitudes, vaccination willingness, and healthcare-seeking behavior

#### 3.3.1 Attitudes toward Mpox prevention and patient care

**Figure 3:**
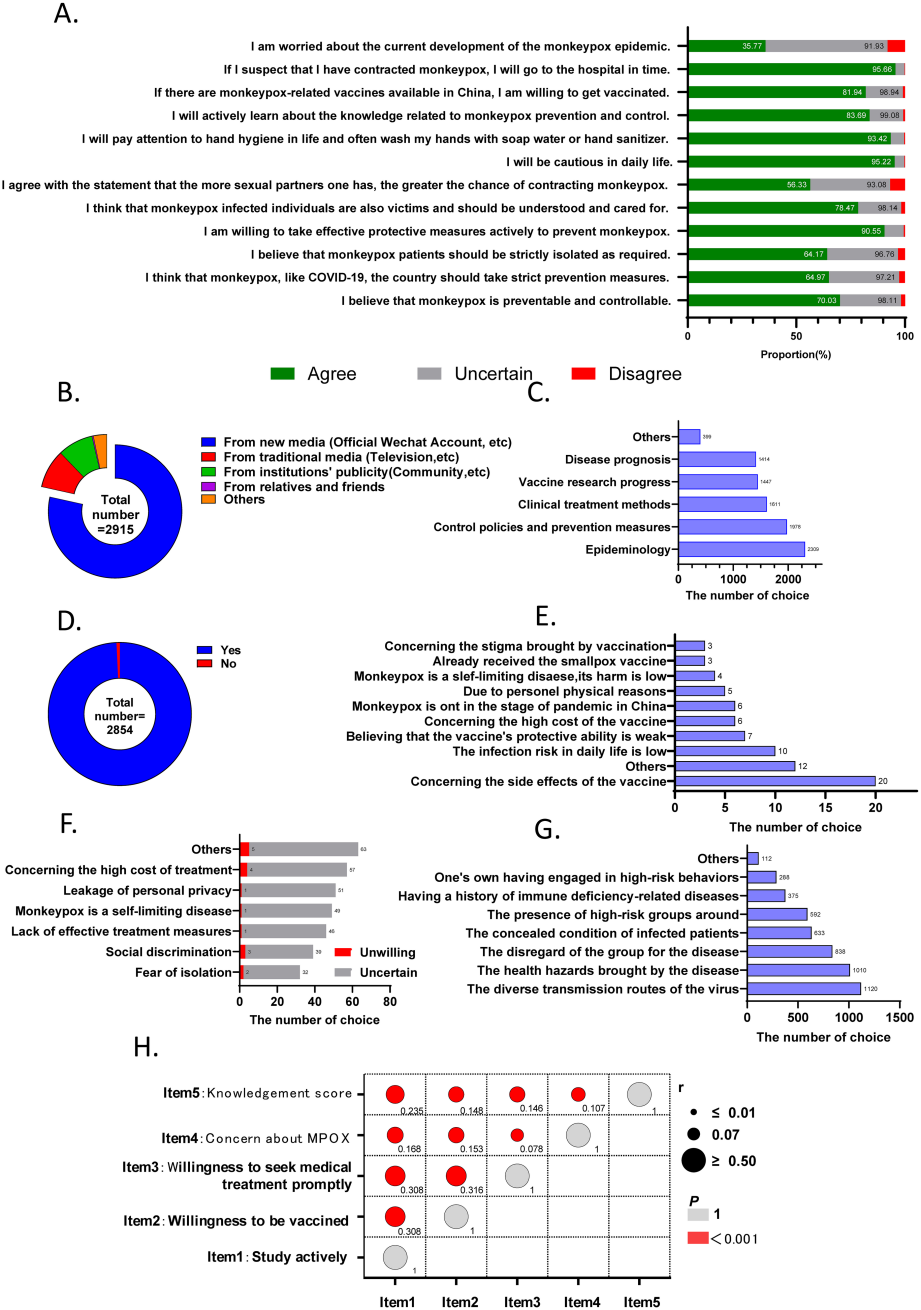
The results on attitude section toward Mpox among 3,483 participants. **(A)** Response distribution for each question in the attitude assessment. **(B)** Sources of Mpox-related knowledge acquisition among 2,915 participants who actively sought information. **(C)** Key topics of interest (multiple-choice) among 2,915 participants who proactively acquired knowledge. **(D)** Proportion of 2,854 vaccine-willing participants who would recommend vaccination to family members. **(E)** Reasons for unwillingness to voluntarily receive vaccination (multiple-choice). **(F)** Reasons for reluctance or uncertainty in seeking medical assistance post-infection (multiple-choice). **(G)** Concerns regarding the current epidemic progression. **(H)** Correlations among knowledge scores, epidemic concerns, vaccination willingness, healthcare-seeking behavior, and proactive knowledge acquisition. The circle sizes represent the r values, and red markers indicate statistical significance (*p* < 0.001).

Attitudes toward Mpox are summarized in Figure 3A. A total of 2,493 (70.03%) participants agreed that Mpox is preventable and controllable, while 2,263 (64.97%) supported the implementation of stringent national preventive measures (32.24% neutral). Additionally, 2,235 (64.17%) endorsed the strict isolation of Mpox patients as mandated by guidelines (32.59% uncertain). A large majority (90.55%) reported willingness to adopt effective protective measures, with 95.22 and 93.42% indicating they would prioritize cleanliness and hand hygiene in daily life, respectively. Furthermore, 78.47% agreed that Mpox patients should be understood and cared for. Regarding perceived transmission risk, 56.33% agreed that “more sexual partners increase Mpox infection risk.”

#### 3.3.2 Proactive information-seeking behaviors

Regarding engagement with Mpox-related knowledge, 2,915 respondents (83.69%) indicated a proactive interest in learning about prevention and control measures. Among these, 79.56% (2,288/2915) primarily obtained information through new media platforms, such as official WeChat accounts. Less than 20% chose to study via traditional media or offline education (Figure 3B). In terms of the specific areas of Mpox knowledge that respondents focused on, epidemiology was the most frequently selected topic (79.21%, 2309/2915), followed by control policies and prevention measures (68.16%, 1978/2915), clinical treatment (55.27%, 1611/2905), vaccine research progress (49.64%,1447/2915), disease prognosis (48.51%, 1414/2915) (Figure 3C).

#### 3.3.3 Vaccination willingness and influencing factors

Vaccination willingness was reported by 2,854 (81.94%) participants, with 592 (17.00%) uncertain and 37 (1.06%) unwilling (Figure 3A). Among those willing to be vaccinated, 99.26% also expressed willingness to recommend vaccination to family members (Figure 3D). The reasons for vaccine hesitancy were illustrated in Figure 3E, with concerns about side effects, other unspecified reasons, and perceived low risk of infection identified as the top three factors. These were followed by apprehensions regarding vaccine efficacy, high cost, and the current domestic epidemiological situation. Notably, when six participants who cited high cost as a concern were asked about their willingness to receive free vaccination, four (66.67%) changed their stance to willing.

Univariable analysis revealed that gender (*p* < 0.001), age (*p* = 0.007), educational level (*p* = 0.033), sexual orientation (*p* < 0.001), and history of sexual intercourse (*p* = 0.004) were associated with willingness to be vaccinated. Multivariable binary logistic analysis indicated that male gender (aOR: 0.665; 95% CI: 0.557–0.793) was negatively associated with vaccination willingness. In contrast, younger age and heterosexual orientation were positive predictors. Participants <20 years (aOR = 2.169, 95% CI: 1.382–3.845) and 20–25 years (aOR = 1.717, 95% CI: 1.094–2.694) had higher willingness than those ≥25 years; heterosexual (aOR = 2.835, 95% CI: 1.861–4.319) and bisexual (aOR = 2.298, 95% CI: 1.284–4.112) individuals were more inclined to accept vaccination than those with other sexual orientations (Table 3).

**Table 3:** The risk factor analysis of vaccine acceptance among 3,483 participants.

Variables	Willingness to be vaccine			Univariate analysis		Multivariate analysis	
	Total	Yes	No or uncertain				
	N (%)	N (%)	N (%)	OR (95%*CI*)	*P*	aOR (95%*CI*)	*P*
Gender							**<0.001**
Male	1,501(43.10)	1,186(79.01)	315(20.99)	0.854(0.785 ~ 0.929)	**<0.001**	0.665(0.557 ~ 0.793)	
Female	1982(56.90)	1,668(84.16)	314(15.84)	Reference		Reference	
Age, years					**0.007**		**0.001**
Age < 20	1842(52.89)	1,535(83.33)	307(16.67)	1.933(1.238 ~ 3.019)	**0.004**	2.169(1.382 ~ 3.845)	**0.001**
20 ≤ age <25	1,537(44.13)	1,244(80.94)	293(19.06)	1.642(1.050 ~ 2.567)	**0.030**	1.717(1.094 ~ 2.694)	**0.019**
Age ≥ 25	104(2.99)	75(72.12)	29(27.89)	Reference		Reference	
Ethnicity							
Han ethnicity	2,954(84.81)	2,432(82.33)	522(17.67)	1.150(0.948 ~ 1.396)	0.159		
Others	529(15.19)	422(79.77)	107(20.23)	Reference			
Educational level					**0.033**		
Below bachelor degree	795(22.83)	656(82.52)	139(17.48)	1.528(1.077 ~ 2.170)	**0.018**		
Bachelor degree	2,455(70.49)	2022(82.36)	433(17.64)	1.512(1.102 ~ 2.075)	**0.010**		
Above bachelor degree	233(6.69)	176(75.54)	57(24.46)	Reference			
Major							
Medical	2001(57.45)	1,645(82.21)	356(17.79)	1.025(0.928 ~ 1.131)	0.633		
Non-medical	1,482(42.55)	1,209(81.58)	273(18.42)	Reference			
Family residence							
Rural	2091(60.03)	1701(81.35)	390(18.65)	0.941(0.843 ~ 1.049)	0.265		
Urban	1,392(39.97)	1,153(82.83)	239(17.17)	Reference			
Sexual orientation					**<0.001**		**<0.001**
Heterosexual	3,165(90.87)	2,616(82.65)	549(17.35)	2.639(1.738 ~ 4.006)	**<0.001**	2.835(1.861 ~ 4.319)	**<0.001**
Homosexual	68(1.95)	52(76.47)	16(23.53)	1.800(0.900 ~ 3.598)	0.096	1.908(0.948 ~ 3.837)	0.070
Bisexual	149(4.28)	121(81.21)	28(18.79)	2.393(1.342 ~ 4.269)	**0.003**	2.298(1.284 ~ 4.112)	**0.005**
Others	101(2.90)	65(64.36)	36(35.64)	Reference		Reference	
History of sexual intercourse							
Yes	340(9.76)	259(76.18)	81(23.32)	0.958(0.928 ~ 0.990)	**0.004**		
No	3,143(90.24)	2,595(82.56)	548(17.44)	Reference			
History of multiple sexual partners							
Yes	48(1.38)	36(75.00)	12(25.00)	0.993(0.982 ~ 1.005)	0.208		
No	3,435(98.62)	2,818(82.04)	617(17.69)	Reference			
History of SIDs							
Yes	18(0.52)	13(72.22)	5(27.78)	0.997(0.989 ~ 1.004)	0.283		
No	3,465(99.48)	2,841(81.99)	624(18.01)	Reference			
Smoking							
Yes	242(6.95)	191(78.93)	51(21.07)	0.985(0.960 ~ 1.010)	0.206		
No	3,241(93.05)	2,663(82.17)	578(17.83)	Reference			
Drinking							
Yes	626(17.97)	510(81.47)	116(18.53)	0.993(0.953 ~ 1.035)	0.735		
No	2,857(82.03)	2,344(82.04)	513(17.69)	Reference			

Hosmer- Lemeshow test: χ^2^ = 1.252, *p* = 0.974. *p* < 0.05 was presented with bold.

#### 3.3.4 Healthcare-seeking behavior and epidemic concerns

A total of 95.66% (3,331/3,483) of participants reported willingness to seek immediate medical care if symptoms suggestive of Mpox appeared, while 142 (4.08%) were uncertain and 9 (0.26%) unwilling (Figure 3A). Reasons for unwillingness to seek care included unspecified other factors (55.56%, 5/9), high medical costs (44.45%, 4/9), and fear of social discrimination (33.33%, 3/9). For participants uncertain about seeking care, the leading reasons were unspecified other factors (44.37%, 63/142), cost concerns (40.14%, 57/142), and privacy breach apprehensions (35.92%, 51/142) (Figure 3F). Additionally, 1,246 respondents (35.77%) reported concerns about the current progression of the Mpox epidemic. The primary factors contributing to these concerns were the diverse transmission routes (89.89%) (Figure 3G).

#### 3.3.5 Correlations among key outcomes

Spearman correlation analysis revealed significant positive correlations between Kscore and proactive information-seeking (*r* = 0.235, *p* < 0.001), Kscore and vaccination willingness (*r* = 0.148, *p* < 0.001), and Kscore and healthcare-seeking behavior (*r* = 0.146, *p* < 0.001). Additionally, proactive information-seeking was positively correlated with vaccination willingness (*r* = 0.308, *p* < 0.001) and healthcare-seeking behavior (*r* = 0.308, *p* < 0.001), and vaccination willingness correlated positively with healthcare-seeking behavior (*r* = 0.316, *p* < 0.001). All pairwise correlations were statistically significant (Figure 3H).

## 4 Discussion

Mpox continues to represent a significant public health challenge due to its unexpected dissemination and introduction across various cities and regions in Chin (25). This study analyzed data from 3,483 college students in northwestern China to assess their disparity of Mpox knowledge and vaccine acceptance.

Regarding knowledge assessment, only four out of 15 questions achieved a correct response rate exceeding 70%. Although most participants demonstrated a fundamental understanding of the etiology and clinical manifestations of Mpox, their knowledge was not incomplete. These findings are consistent with those reported in previous research (20). The majority of Mpox patients would present with symptoms such as fever, skin lesion and lymphadenopathy whereas a minority remain asymptomatic (6, 26, 27). Most respondents knew the virus agent and nonspecific symptoms after Mpox infection. However, fewer than 70% were aware of lymphadenopathy in the groin or other regions among infected individual, which serves as a distinguishing clinical feature when compared to other viral rash illnesses (26, 28).

An insufficient understanding of virus transmission, clinical management, and preventive strategies was identified among respondents. Only less than 50% recognized that the entire population is susceptible to Mpox and it is infection disease classification in China. This finding aligns with their knowledge regarding viral transmission and preventive measures. The primary modes of transmission during the recent outbreak were animal-to-human or human-to-human physical close contact interaction (29). MPXV can be detected in skin, nasal, oral, and anal secretions, with viral shedding from the upper respiratory tract persisting for over 3 weeks (30). Besides, high concentrations of virus were identified in aerosols from a hospital (31). MPXV can still be transmitted via droplets or aerosols, though rare outbreaks have been reported. Additionally, infectious virus particles can be detected on environmental surfaces such as door handles, walls, and floors, indicating the long presence of viral particles in the surroundings (32–35). The proportion of correct responses related to transmission through general close contact was higher than via droplet and towels with infected individuals. Notably, only 36.29% knew contagion of scabs, despite evidence that MPXV can be transmitted through direct contact with scabs (36, 37). These findings suggest that we should enhance public education on disease transmission routes with more detailed and specific guidance in subsequent efforts. This is crucial to prevent the virus from going unrecognized by the general public in later stages, which could lead to undetected community transmission.

The study revealed variable Kscore distributions across subgroups, while similar patterns have been documented in previous research (38, 39). Further analysis indicated that females were more likely to achieve higher knowledge scores, which is consistent with a cross-sectional study involving medical students from 27 countries (40). Students majoring in medicine also demonstrated a tendency toward higher Kscore, corroborating results from other studies (20, 39, 41). However, contrasting finding was reported in southern China, medical students exhibited relatively limited knowledge level and less enthusiasm for proactive studying compared to non-medical counterparts (19). This discrepancy may be attributed to differences in study populations and geographic contexts. Urban residents were more likely to get higher Kscore compared to those from rural. The urban–rural disparity in health education and infectious disease control is well-documented (42). In the western region, economic conditions are comparatively poorer, and there exist notable disparities in primary healthcare access between urban and rural areas. Additionally, the widespread distribution of rural communities and the prevalence of migrant workers contribute to a lack of relevant information and healthcare for students in these areas, often due to limited familial or social networks (43). Han ethnicity tended to achieve higher Kscore may be related to a more enlightened and rational perspective on issues pertaining to sexuality. Furthermore, alcohol consumption was negatively associated with higher Kscore. Given the known adverse health effects of alcohol, alcohol consumers may be less attentive to health-related issues and thus acquire knowledge primarily through passive exposure within their social environment. The aforementioned results delineate the demographic profile of high-risk population, education initiatives should be implemented, with male university students who drink alcohol being the prime target for focused attention.

Most participants expressed a positive attitude towards the domestic epidemic. Following the international epidemic outbreak, the Chinese government implemented timely and strict control measures - issuing epidemic bulletins, enhancing entry-exit quarantine and formulating guidelines, which effectively curbed the diseases rapid spread and local prevalence (12). However, only 64.97 and 64.17% showed agreement with strict prevention action and isolation of patients. According to *Technical guidelines for monkeypox control in China* (2022 version), suspected and confirmed cases should be transported to designated professional infectious disease treatment institutions for strict isolation. The result shows most students were not acquainted with the prevention and control policies. Interestingly, high agreement was observed about personal protection. Most respondents would take effective protective measures and learn related knowledge via new media. This finding provides important insights for us to select the most effective health education approaches in the future.

More than one third students, a proportion similar to that observed in other Chinese area (20, 44), shown a concern about the development of Mpox pandemic. Basic knowledge regarding the treatment and prevention of Mpox still attracted public attention on the internet and Gansu was the top 10 province concerned about issues (45). In our study, virus transmission and prognosis were the most concerned aspects. Besides, most respondents would seek medical help after infected. These results underline the worry to their own health, showing a positive sign for the implementation of the prevention policies. It also suggests that in subsequent communications, we can enhance students’ prevention awareness by emphasizing the diversity of the virus’s transmission routes and its potential adverse outcomes.

61.18% of participants believed that correct condom use has full protective capacity, a misconception potentially stemming from persistent misinformation portraying Mpox as a novel sexually transmitted infection (46). The predominance of research focusing on MSM or HIV-infected, which titles containing “sex with” “MSM,” etc., might lead to misperceptions among the general population who lack the capacity to critically interpret these findings. Such inadequate awareness could adversely affect disease control efforts during potential outbreaks (47). The rapid spread of misinformation on the internet, progresses to proliferation of stigmatization and health inequity (23, 48, 49). 78.47% of respondents agreed that Mpox patients should be understand and cared for, which indicate a correct understanding and perception of diseases, but there were still one fifth conversed. It is imperative to make eliminating these misconceptions a top priority. Strict information verification, should be implemented by government agencies and media platforms. Health education campaigns should avoid using aforementioned term as titles and instruct personal protective measures among college students in Western China.

Vaccination was the most cost-effective method for preventing virus spread. 81.94% of students shown willingness to be vaccinated if it was available in China, and most of them were willing to recommend to family members. This proportion is similar to prior research in northwestern China (50), higher than the students in north and northeast China (20) and around the world (51). The hesitancy rate was 56.13% toward the Mpox vaccine among students in southwest China due to concerns about vaccine safety (52). The same reason ranked first in our study. Effectiveness of a counseling intervention can increase vaccination acceptance and uptake (53). It implys that we should strengthen health education on vaccination safety if vaccine was available. Further analysis suggested the associated factor of vaccine acceptance only including gender, age and sexual orientation. The Female shown a higher willingness than male, which was similar to previous study (20). The younger age population shown higher willingness of vaccination in our study, which was also observed in other survey (20, 54). This might attribute to higher confidence of new medical technology among younger population. A survey conducted in Hongkong indicated the bisexual and heterosexual or others population shown higher vaccine acceptance than gay population (55), Which is different to our result. Sexual orientation is important privacy because of various reason and rarely people willing to make it public thus weaken the application of this indicator in public health. More research should be applied to this field. Nevertheless, we still argue that young male college participants could be prioritized in vaccine promotion efforts.

Correlation analysis showed that the correlation between Kscore and proactive information-seeking was relatively stronger, suggesting that students with a higher initial knowledge reserve may more likely to form a positive cycle of “active learning.” The correlation between proactive information-seeking and both vaccination willingness and healthcare-seeking behavior was even stronger, highlighting the importance of “optimizing information dissemination channels” to enhance the willingness of vaccination and healthcare-seeking. It should be noted that all correlation coefficients among the variables in this study were of low to moderate strength, indicating that the relationships between variables are modulated by multiple factors. Future studies can further clarify the functional pathways among these variables through mediation effect analysis, so as to provide a basis for more targeted interventions.

### 4.1 Limitation

This was a quick cross-sectional survey based on self-reported questionnaire. The convenience sampling method could not avoid the selection bias. The higher proportion of medical students might be due to their greater attention to the progress related to their own field leading to a higher response rate. This may not reflect the actual situation of all college students in northwest China. We acknowledge that the lack of identifying data (e.g., school or grades) prevents us from analyzing whether these factors influence Mpox knowledge or vaccination willingness. Thus, the result should be interpreted with caution when generalized to all students in the region. In addition, in the questionnaire survey research, it is inevitable that recall bias and social desirability bias. Regarding issues such as smoking, drinking, sexual life, and feelings of shame and social acceptance, some participants may have made choices that align with mainstream social ideology. Future studies should expand their coverage and combine self-reported questionnaires with objective data to reduce the impact of bias.

## 5 Conclusion

Our study was the first research conducted among college students in northwest China to evaluate the knowledge, attitude and vaccine acceptance. The results showed a lack of Mpox-related knowledge but a higher personal protection and vaccine acceptance in surveyed population. Educational initiatives could be implemented via WeChat, covering key topics such as transmission routes, vaccine safety, and stigma reduction. It is advisable to target male freshmen with unhealthy lifestyles as a key group for health education.

## Data Availability

The raw data supporting the conclusions of this article will be made available by the authors, without undue reservation.

## Footnotes

1
https://www.powercx.com/product
