# Supplementary material for: Disparity in Mpox awareness and vaccine hesitancy: a cross-sectional study of 3,483 university students in Northwest China
Source: Front Public Health. 2025 Nov 27;13:1657497. doi: 10.3389/fpubh.2025.1657497 (PMC12695810; doi:10.3389/fpubh.2025.1657497)
Supplement: Supplementary file 1 [file Table_1.docx]

**Supplemental table1: The content of questionnaire**

**The questionnaire of MPOX knowledge and attitude among college students**

Please complete the following reading!

Hello! Dear volunteers. This study was conducted by the Centers for Disease Control and Prevention (CDC) to understand college students’ knowledge and attitudes toward the MPOX. **To allay your concerns, information that may be related to identity verification, such as ID numbers, student numbers, schools attended, and grades, will not be collected in this survey.** All data in this study are only used for scientific research to provide basic information for epidemic prevention and control. No other units or individuals will know your relevant information.

**Q1. Are you a college student?**

(1) Yes (2)No

**Q2. Have you known the above and volunteered to fill in the questionnaire?**

(1) Yes (2)No

**Q3. What is the date today?**

( - - ) (yyyy-mm-dd)

Thank you for your participation. Please answer the following questions carefully.

**Part 1 *Demographic information***

**1.Gender**:

(1) Male (2) Female

**2.Age**: ( ) years old.

**3.Ethnicity**: (1) Han ethnicity (2) Others

**4.Major:** (1) Medical major (2) Non-medical major

**5.Sexual orientation**: (1) Heterosexual (2) Homosexual (3) Bisexual (4) Others

**6.Educational level**: (1) Junior college (2) Bachelor's degree (3) Graduate degree

**7.Family residence:** (1) Rural (2) Urban

**8.Have you had sexual intercourse before:** (1) Yes (2) No

**9.Have you had multiple sexual partners** (≥2): (1) Yes (2) No

**10.Have you ever been infected with the following diseases**: (1) HIV (2) Genital warts, gonorrhea, syphilis, etc. (sexually transmitted diseases) (3). Both (4). Neither

**11.Smoking**: (1) Yes (2) No

**12.Drinking**: (1) Yes (2) No

**Q4. What time is it now?**

( : ) (24-hour system)

**Part 2 *Knowledge part***

**Note: The correct answers were painted in green. Added 1 score when chose the correct answer. *This note was invisible to participants.***

**1. Monkeypox is caused by the monkeypox virus (MPXV).**

(1) Yes (2) No (3) Don't know

**2. In China, monkeypox is managed as a Class B infectious disease.**

(1) Yes (2) No (3) Don't know

**3. People can be infected with monkeypox through close contact with monkeypox patients or animals.**

(1) Yes (2) No (3) Don't know

**4. The monkeypox virus can be transmitted through close-range droplet transmission.**

(1) Yes (2) No (3) Don't know

**5. Sharing towels, toothbrushes, tableware, etc. with monkeypox patients can lead to infection with monkeypox.**

(1) Yes (2) No (3) Don't know

**6. The general population is susceptible to monkeypox.**

(1) Yes (2) No (3) Don't know

**7. The male homosexual community is a high-risk group for monkeypox infection in China.**

(1) Yes (2) No (3) Don't know

**8. After being infected with monkeypox, symptoms such as chills, fever, headache, back pain, and muscle pain can occur.**

(1) Yes (2) No (3) Don't know

**9. After being infected with monkeypox, superficial lymph nodes such as those in the armpits and groin can become swollen.**

(1) Yes (2) No (3) Don't know

**10. After being infected with monkeypox, skin rashes can occur.**

(1) Yes (2) No (3) Don't know

**11. When the skin lesions of monkeypox patients form scabs, they are no longer contagious.**

(1) Yes (2) No (3) Don't know

**12. Correct use of condoms can completely prevent the risk of monkeypox infection.**

(1) Yes (2) No (3) Don't know

**13. Having received the smallpox vaccine provides a certain degree of cross-protection against the monkeypox virus.**

(1) Yes (2) No (3) Don't know

**14. Currently, there are no specific treatment drugs for monkeypox.**

(1) Yes (2) No (3) Don't know

**15. Currently, there are no vaccines available in mainland China to prevent monkeypox.**

(1) Yes (2) No (3) Don't know

**Part 3 *Attitude part***

**1. I believe that monkeypox is preventable and controllable.**

(1) Disagree (2) Uncertain (3) Agree

**2. I think that monkeypox, like COVID-19, the country should take strict prevention measures.**

(1) Disagree (2) Uncertain (3) Agree

**3. I believe that monkeypox patients should be strictly isolated as required.**

(1) Disagree (2) Uncertain (3) Agree

**4. I am willing to take effective protective measures actively to prevent monkeypox.**

(1) Disagree (2) Uncertain (3) Agree

**5. I think that monkeypox infected individuals are also victims and should be understood and cared for.**

(1) Disagree (2) Uncertain (3) Agree

**6. I agree with the statement that "the more sexual partners one has, the greater the chance of contracting monkeypox".**

(1) Disagree (2) Uncertain (3) Agree

**7. I will be cautious in daily life.**

(1) Disagree (2) Uncertain (3) Agree

**8. I will pay attention to hand hygiene in life and often wash my hands with soap water or hand sanitizer.**

(1) Disagree (2) Uncertain (3) Agree

**9. I will actively learn about the knowledge related to monkeypox prevention and control.**

(1) Disagree (2) Uncertain (3) Agree

***If the “Agree” was chosen, it will jump to the item 9.1 and 9.2***

**9.1 Please tell me which aspect of monkeypox knowledge you are focusing on****（Multiply choice）:**

1. Epidemic situation
2. Control policies and prevention measures
3. Clinical treatment methods
4. Vaccine research progress
5. Disease prognosis
6. Others

**9.2 Please tell me your main source of knowledge acquisition:**

1. Official WeChat Account/Video, Weibo, Douyin, Kuaishou and other new media
2. Television, news, radio and other traditional media
3. Community, village, school, hospital and other institutions' publicity
4. From relatives and friends
5. Others

**10. If there are monkeypox-related vaccines available in China, I am willing to get vaccinated.**

(1) Disagree (2) Uncertain (3) Agree

***If the “Disagree” was chosen, it will jump to the item 10.1.***

***If the “Agree” was chosen, it will jump to the item 10.2.***

**10.1 The reason why you don't want to get vaccinated is（Multiply choice）：**

1. Concerning the side effects of the vaccine
2. Believing that the vaccine's protective ability is weak
3. The infection risk in daily life is low
4. Concerning the high cost of the vaccine

4.1 **If the vaccination is free, are you willing to get vaccinated. (1). Yes (2). No**

1. Monkeypox is not in the stage of pandemic in China
2. Due to physical reasons, it is not suitable for vaccination
3. Monkeypox is a self-limiting disease, its harm is low
4. I have already received the smallpox vaccine
5. Concerning the stigma brought by vaccination, such as being labeled as a member of a certain group
6. Others

**10.2 Are you willing to recommend it to your family and friends for vaccination?**

(1). Yes (2). No

**11. If I suspect that I have contracted monkeypox, I will go to the hospital in time.**

(1) Disagree (2) Uncertain (3) Agree

***If the “Disagree” or “Uncertain” were chosen, it will jump to the item 11.1.***

**11.1 The reasons why you don't want to seek medical treatment（Multiply choice）.**

- 1. Social discrimination
  2. Monkeypox is a self-limiting disease
  3. Leakage of personal privacy
  4. Concerning the high cost of treatment
  5. Lack of effective treatment measures
  6. Fear of isolation
  7. Others

**12.** **I am worried about the current development of the monkeypox epidemic.**

(1) Disagree (2) Uncertain (3) Agree

***If the “Agree” was chosen, it will jump to the item 12.1.***

**12.1 Could you please tell me the main reason for your concern?**

1. The diverse transmission routes of the virus
2. The health hazards brought by the disease
3. The disregard of the group for the disease
4. The presence of high-risk groups around
5. One's own having engaged in high-risk behaviors
6. Having a history of immune deficiency-related diseases
7. The concealed condition of infected patients
8. Other

Thank you for volunteer in this survey.


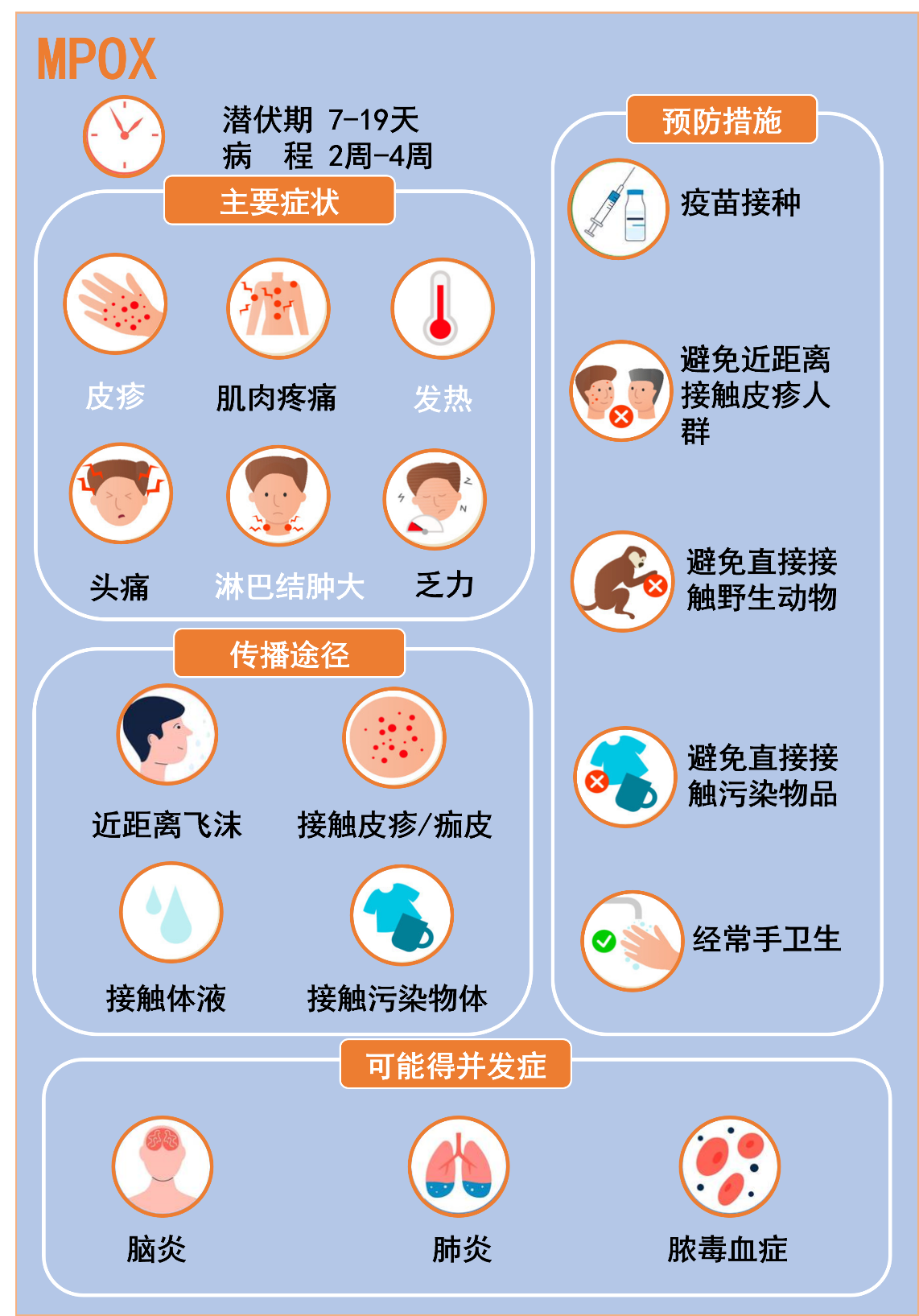
**Note: The concise health promotio** **n page will only be displayed after the answer is completed.**
